# Safety of vaginal breech delivery following an unsuccessful external cephalic version: a comparative study

**DOI:** 10.1007/s00404-024-07873-9

**Published:** 2024-12-26

**Authors:** Danit Aviv, Amir Weintraub, Gal Issakov, Yael Pasternak, Rachel Griffin, Tzipora Shochat, Miriam Lopian, Yael Yekel, Sharon Perlman

**Affiliations:** 1 Adelson School of Medicine, Ariel University, Ariel, Israel; 2 Department of Obstetrics and Gynecology, Laniado Medical Center, Divrei Hayim 16, Netanya, Israel; 3 Tel Aviv University School of Medicine, Tel Aviv, Israel; 4 Rabin Medical Center, The Helen Schneider Women’s Hospital, Petah Tikva, Israel

**Keywords:** External cephalic version, Vaginal breech delivery, Trial of labor, Cesarean delivery

## Abstract

**Objective:**

To determine whether patients undergoing a trial of labor with a breech presentation following a failed attempt of external cephalic version (ECV) are at increased risk of adverse maternal and neonatal outcomes.

**Methods:**

This retrospective cohort study was conducted at a single university-affiliated medical center. The study group comprised women with singleton pregnancies at term, categorized into three groups: those who underwent a failed external cephalic version (ECV) and subsequently attempted a trial of breech delivery (Breech-failed-ECV group), those who attempted an assisted vaginal breech delivery without a prior ECV attempt (Breech-no-ECV group), and those with vertex presentation following a successful ECV (Vertex-ECV). The primary outcome measured was the mode of delivery. Secondary outcomes included adverse maternal and neonatal outcomes.

**Results:**

The study group consisted of 229 patients who attempted a vaginal delivery during the study period following a diagnosis of non-cephalic presentation at term. There were 42 women in the Breech failed-ECV group, 102 in the Breech-no-ECV group, and 85 in the Vertex-ECV group. Among patients undergoing a trial of labor with a breech presentation, there were no significant differences in successful vaginal delivery rates between those who had an attempted ECV and those who did not (80.39% vs. 80.95%, p > 0.05), nor in the rate of adverse maternal or neonatal outcomes between the groups. However, the Vertex-ECV were more likely to have a vaginal delivery (91.78 vs 80.56%, p = 0.03) and less likely to experience adverse neonatal outcomes, including meconium-stained amniotic fluid, non-reassuring fetal heart rate (NRFHR), compared to those who underwent labor with a breech presentation (p < 0.05).

**Conclusions:**

A failed external cephalic version does not adversely affect maternal or neonatal outcomes in patients undergoing a trial of labor with a breech presentation and meet the criteria of our study.

**Supplementary Information:**

The online version contains supplementary material available at 10.1007/s00404-024-07873-9.

## What does this study add to the clinical work

**Table:** 

A failed external cephalic version does not worsen assisted breech delivery outcomes.	

## Introduction

The optimal method of delivery for a fetus in a breech presentation has always been a matter of debate. Even before the publication of the Term Breech Trial, elective cesarean section was commonly performed for breech presentation at term due to concerns about the risks associated with vaginal breech delivery. However, the findings from the Term Breech Trial in 2000 [1], which established the safety benefits of elective cesarean delivery over a trial of labor, led to a shift in obstetric practice in favor of almost routine elective cesarean delivery in these cases. This resulted in a significant reduction in the rate of vaginal breech deliveries, particularly in resource-rich countries, along with a corresponding rise in the rate of elective cesarean deliveries for this indication.

The increasing rates of cesarean delivery, with their associated short and long-term morbidity, prompted the introduction of the External Cephalic Version (ECV) into obstetric practice. Success rates for attempted ECV range from 49–72% [2, 3], and ultimately, this practice offers a practical alternative to elective cesarean delivery and significantly increases the chances of successful vaginal delivery [4].

Although attempted ECV is generally considered safe, there have been reports of placental abruption and non-reassuring fetal heart rate (NRFHR) tracings requiring emergency cesarean delivery after an ECV attempt [5, 6]. Furthermore, a trial of labor after a successful ECV is still associated with a higher rate of unplanned cesarean delivery, operative vaginal delivery, and adverse neonatal outcomes [7–9] compared to labor in a spontaneous vertex presentation.

In recent years, there has been a renewed interest in assisted breech delivery, particularly among certain populations with a strong preference for vaginal birth. This shift in obstetric practice is supported by a prospective study by Goffinet et al. [10], which demonstrated the safety of vaginal breech delivery in carefully selected patients who met strict criteria, including normal pelvimetry, frank breech presentation, and estimated fetal weight between 2500 and 3800 g, as well as additional factor.

Despite these encouraging results, the recommendation of the American College of Obstetricians and Gynecologists (ACOG) and the Royal College of Obstetricians and Gynecologists (RCOG) [11, 12] is to first consider a trial of external cephalic version (ECV) before considering the option of a trial of labor in a breech presentation. That said, there are still select groups of women who wish to pursue a trial of a breech vaginal delivery without an attempted ECV, motivated by a philosophy of natural childbirth or concerns regarding safety, associated pain, and potential complications of an ECV that could necessitate a cesarean section [6, 13].

This study aims to assess whether patients attempting labor with a breech presentation after an unsuccessful external cephalic version (ECV) face higher risks of adverse maternal and neonatal outcomes compared to those attempting labor in a breech presentation without a prior ECV. The results could help guide counseling for patients with breech presentations in the late third trimester who wish to avoid cesarean delivery or an ECV attempt.

## Methods

A retrospective cohort study was conducted at a single university-affiliated medical center with approximately 7000 deliveries annually between 2015 and 2022. Patients were included in the study if they had a breech presentation at term and wanted to avoid cesarean delivery. The study group of interest was comprised of patients who had an ECV, which was unsuccessful, but subsequently opted for a trial of labor with a breech presentation (Breech-failed-ECV group). Outcomes were compared to those of two control groups. The first group was comprised of women who underwent a trial of breech delivery without a prior ECV attempt (referred to as the Breech-no-ECV- group), and the second group was comprised of patients who had a successful ECV followed by a trial of labor in a vertex presentation (Vertex-ECV group). Further analyses were performed comparing the outcomes of patients who underwent a trial of labor in vertex presentation (Vertex-ECV) to those who underwent a trial of labor in breech presentation (Breech-failed- ECV group combined with Breech-no-ECV group).

The inclusion criteria for planned vaginal breech delivery, according to our institution’s protocol, include the following: a singleton fetus, gestational age of at least 37 weeks, at least one prior vaginal delivery, an estimated fetal weight lower than the previous neonatal birth weight and below 4000 grams, frank or complete breech presentation, and a neutral or flexed head position on sonographic assessment. The exclusion criteria include fetal anomalies, severe fetal growth restriction, more than one prior cesarean section, contraindications to vaginal delivery, estimated fetal weight exceeding 4000 grams, and footling presentation. Additionally, a skilled attending obstetrician must be present during the delivery. All cases in the Breech-failed-ECV and Breech-no-ECV groups met these criteria.

To minimize bias related to parity, the control group (Vertex-ECV) was comprised of women who had at least one prior vaginal delivery.

The primary outcome was the mode of delivery. Secondary outcomes included non-reassuring fetal heart rate (NRFHR), meconium-stained amniotic fluid, cord prolapse, placental abruption, umbilical artery pH < 7.1, 5-min Apgar score < 7, neonatal intensive care unit (NICU) admission, and adverse maternal outcomes such as postpartum hemorrhage (PPH), cesarean section surgical site infection, thromboembolic complications, and deep vein thrombosis.

### Statistical analysis

Maternal demographic data and obstetrical outcomes were obtained from the medical records. Continuous variables were presented as either mean ± standard deviation (SD) or median with interquartile range (IQR), depending on their distribution. Categorical variables were expressed as counts and percentages (N, %). The Kolmogorov–Smirnov test assessed the normality of continuous variables. For overall comparisons among the three study groups, analysis of variance (ANOVA) was employed for normally distributed continuous variables, and the Kruskal–Wallis test was used for non-normally distributed variables. Fisher’s exact test was utilized for categorical variables.

Pairwise comparisons between the study groups were conducted using the appropriate statistical tests. Specifically, t-tests were employed to compare normally distributed continuous variables, Mann–Whitney tests were used for non-normally distributed continuous variables, and Fisher’s exact test was applied for categorical variables. Odds ratios were calculated to assess the likelihood of specific outcomes between different study groups.

The statistical analysis was conducted using SAS Software, Version 9.4. Statistical significance was set at a two-sided p-value of less than 0.05, indicating a statistically significant difference between groups.

## Results

Between 2015 and 2022, there were 58,308 deliveries at our medical center. Of those, 229 (0.003%) were included in our study cohort. There were 42 women (18.3%) in the Breech-failed-ECV group, 102 (44.5%) in the Breech-no-ECV g group, and 85 (37.1%) in the Vertex-ECV group.

There were no clinically significant differences among the three groups regarding maternal age, gestational age at delivery, parity, previous cesarean section, or gestational diabetes. The Vertex-ECV group was significantly more likely to have undergone labor induction or augmentation compared to the Breech-failed-ECV group and Breech-no ECV group (30.59% vs. 5.88% vs. 4.79%, p < 0.001), and (36.47% vs. 16.67% vs. 23.8%, p = 0.009). They also delivered neonates with a higher mean birthweight than the breech groups (3448 ± 410 g (g) vs. 3059 ± 398 for Breech-failed-ECV and 3142 ± 485g for Breech-no-ECV, p = 0.0001) (Table 1).

**Table 1:** Demographic characteristics of the study group

	Vertex-ECV *n* = 85	Breech-No – ECV *n* = 102	Breech-failed- ECV *N* = 42​	*P* value
Maternal Age​ (years)	32.87​	32.69​	32.36​	0.89​
Gestational age​ (weeks)	39.64​	39.44​	39​	0.028​
Parity​	4.86​	4.96​	4.57​	0.56​
Previous CS %​	17.65​	6.86​	11.9​	0.069​
GDM/PGDM %​	17.65​	9.8 ​	9.52​	0.52​
Birthweight (gr)​	3448​	3142​	3059​	0.0001​

*CS* cesarean section, *GDM* gestational diabetes mellitus, *PGDM* pregestational diabetes mellitus

With regards to the primary outcome of the mode of delivery, there were no statistically significant differences in the rate of successful vaginal delivery between Breech-failed -ECV compared to Breech-no-ECV (80.9% vs. 80.4% p = 0.98). However, patients in the Vertex- ECV group were significantly more likely to achieve a vaginal delivery than Breech no ECV group (91.76% vs 80.39% respectively = 0.03).

With regard to secondary outcomes, patients in the Breech-failed-ECV group exhibited a reduced risk of meconium-stained amniotic fluid (7.1% vs. 26.4%, p = 0.01) compared to the Breech-no-ECV group. However, this group also showed a higher risk of NICU admission and non-reassuring fetal heart rate (NRFHR) compared to the Vertex-ECV group (7.14% vs. 0%, p = 0.03; 16.6% vs. 3.53%, p = 0.01). Importantly, while these associations were significant in the initial analysis, logistic regression adjustment revealed that the NICU admission was no longer statistically significant.

Patients in the Breech-no-ECV group had a higher risk of Meconium-stained amniotic fluid than Vertex-ECV (26,4% vs 9.41%, p = 0.004). There were no statistically significant differences between the other findings (Table 2).

**Table 2:** Maternal and neonatal outcomes in the three study groups (p-values overall)

	Vertex-ECV *n* = 85 (*N*, %)	BREECH -no- ECV *n* = 102 (*N* %)	BREECH-failed- ECV *n* = 42 (*N*, %)	*P* value
​​ Induction of labor	26(30.59)	6 (5.88)	2 (4.79)	< 0.001*
Augmentation of labor	31 (36.47)	17 (16.67)	10 (23.8)	0.009**
Vaginal delivery	78 (91.78)	82 (80.39)	34 (80.95)	0.06***
Cesarean section	5 (5.88)	18 (17.65)	8 (19.05)	0.02^†^
Operative vaginal delivery	2 (2.35)	2 (1.96)	0 (0)	1
NRFHR	3 (3.53)	11 (10.78)	7 (16.6)	0.03^§^
Meconium-stained amniotic fluid	8 (9.41)	27 (26.47)	3 (7.14)	0.002^‡^
Prolapse of cord	0 (0)	3 (2.94)	0 (0)	0.3
Placental abruption	0 (0)	4 (3.92)	0 (0)	0.14
pH < 7.1	0 (0)	1 (0.98)	2 (4.76)	0.3
APGAR score < 7	0 (0)	0 (0)	0 (0)	N
NICU admission	0 (0)	4 (3.92)	3 (7.14)	0.04^#^
PPH	4 (4.71)	7 (6.86)	2 (4.76)	0.93
Adverse maternal outcome	4 (4.71)	6 (5.88)	2 (4.76)	0.927

*NRFHR* nonreassuring fetal heart rate, *NICU* neonatal intensive care unit, *PPH* postpartum hemorrhage

*Significant for VX-ECV vs. Breech failed ECV (*p* < 0.001) and for ECV-VX vs. Breech no ECV (*p* < 0.001)

**Significant for ECV-VX vs. Breech no ECV (*p* = 0.002)

***Significant for ECV-VX vs. Breech no ECV (*p* = 0.03)

^†^Significant for VX-ECV vs. Breech failed ECV (*p* = 0.03), and for ECV-VX vs. Breech no ECV (*p* = 0.02)

^§^Significant for ECV-VX vs. Breech failed ECV (*p* = 0.01)

^‡^Significant for ECV-VX vs. Breech no ECV (*p* = 0.004) and Breech no ECV vs. Breech failed ECV (*p* = 0.01)

^#^Significant for ECV-VX vs. Breech failed ECV (*p* = 0.03)

### Analyses based on fetal presentation during labor

When performing the analyses based on fetal presentation at delivery, compared to those who underwent a trial of labor in a breech presentation (Breech-failed-ECV and Breech-no-ECV), patients in the Vertex-ECV group were more likely to achieve a successful vaginal delivery (91.76%% vs. 80.56%, *p* = 0.03). They were also less likely to have meconium-stained amniotic fluid (9.41% vs. 20.8%, *p* = 0.03) and a non-reassuring fetal heart rate (NRFHR) tracing (3.5% vs. 12.5%, *p* = 0.04) compared to the Breech presentation group. However, these differences were not associated with adverse neonatal outcomes, as there were no differences between groups in the rates of APGAR scores below seven at five minutes, umbilical artery pH < 7.1, and NICU admission (Table 3).

**Table 3:** Maternal and neonatal outcomes: Vertex-ECV vs. all breach

	Vertex-ECV *N* = 85​	All Breech *N* = 144​	OR [95% CI]	*P* value​
​​ Induction	26 (30.59​)	8 (5.56​)	7.1 (3.1–16.4)	< 0.001​
Augmentation	31 (36.47​)	27 (18.75​)	2.4 (1.3–4.5)	0.003
Vaginal delivery	78 (91.76​)	116 (80.56​)	2.5 (1.08–6.04)	0.03
Cesarean section	5 (5.88)	26 (18.06)	0.3 (0.1–0.8)	0.016
Instrumental delivery	2 (2.35)	2 (1.38)	1.7 (0.28–10.1)	0.55
NRFHR	3 (3.53)	18 (12.5​)	0.29 (0.08–0.94)	0.04​
Meconium-stained amniotic fluid	8 (9.41)	30 (20.83​)	0.4 (0.18–0.93)	0.03
Prolapse of cord	0 (0)	3 (2.08​)	0.23 (0.012–4.7)	0.34
Placental abruption	0 (0)	4 (2.78​)	0.18 (0.01–3.49)	0.25
PH < 7.1	0 (0)	3 (2.08​)	0.4 (0.01–16.9)	0.67
APGAR score < 7	0 (0)	0 (0)		N​
NICU admission	0 (0)	7 (4.86​)	0.1 (0.006–1.8)	0.12
PPH	4 (4.71)	9 (6.25​)	0.78 (0.24–2.51)	0.68
Adverse maternal outcome	4 (4.71)	8 (5.56​)	0.88 (0.27—2.88)	0.84

## Discussion

### Principal findings

Our findings suggest that a successful ECV reduced the rate of cesarean deliveries without adversely affecting maternal or neonatal outcomes, and a failed ECV attempt did not increase the rate of C-sections when compared to women who attempted to deliver a fetus in a breech delivery at term without undergoing ECV. Our results provide valuable data for the prenatal counseling of women with a fetus in a breech presentation at term who are debating the mode of delivery. We demonstrate the advantages of attempting an ECV, which, if successful, significantly increases the rate of vaginal deliveries and, if unsuccessful, doesn’t reduce the chance of vaginal delivery nor put the neonate at increased risk of adverse outcomes, compared to those who did not attempt an ECV. The option to proceed with attempted vaginal breech delivery if ECV failed was not associated with an increased rate of adverse obstetric outcomes.

The results of the Term Breech Trial have led to a significant increase in the cesarean delivery rate for breech presentations. This has almost ended vaginal breech deliveries for the period following this trial [1], even though we know cesarean deliveries are not without risk, including immediate surgical complications as well as an increased rate of placenta accreta in subsequent pregnancies and potential implications for future family planning [14].

To counter these concerns, an attempted External Cephalic Version (ECV) emerged as a promising alternative to avoid cesarean delivery in patients with a breech presentation. However, attempted vaginal breach deliveries, even after a successful ECV, are still associated with increased risks, namely, unplanned operative deliveries and poorer neonatal outcomes in nulliparous and multiparous women compared to those laboring with a spontaneous vertex presentation [7–9]. These findings have raised concerns about whether a trial of ECV, regardless of its success, may result in suboptimal repositioning of the presenting part, predisposing to reduced chances of achieving a successful vaginal delivery.

Recently, there has been a notable increase in the number of women expressing interest in and choosing planned vaginal breech delivery as an alternative to cesarean section. This highlights the need for more current research to evaluate the safety and effectiveness of vaginal breech delivery in various populations. In a cohort study analyzing women with a singleton breech presentation, it was found that elevated BMI did not increase perinatal morbidity during vaginal deliveries. However, cesarean delivery rates were significantly higher in overweight and obese women (43.9% compared to 29.3%)[15]. Additionally, it aims to determine whether there is a role for attempting an external cephalic version (ECV) for patients and physicians interested in pursuing a breech delivery. This data would enable physicians to provide evidence-based counseling to women with a breech presentation, facilitating shared and patient-centered decision-making for those considering these deliveries. It would also help determine the optimal balance between external cephalic version (ECV) and planned vaginal breech deliveries, guiding evidence-based care for patients motivated to pursue a vaginal delivery.

Our findings demonstrate that having ECV does not compromise the chances of achieving a successful vaginal delivery compared to not having an ECV at all, and an ECV attempt itself does not predispose to increased risks of adverse outcomes. Our findings contrast with those of Blayala et al. [16], which included 183,323 cases of women with breech presentation and revealed that failure of ECV was associated with higher rates of cesarean deliveries, and increased perinatal and obstetric complications (lower rate of vaginal deliveries, higher rate of NRFHR and cesarean sections). The discrepancies observed in our results may be attributed to several factors. Notably, our study did not include nulliparous women with breech presentations, which may influence outcomes, as women with previous deliveries may respond differently to external cephalic version (ECV) and vaginal breech delivery. In addition, variations in medical protocols may also impact the results.

Furthermore, when we performed the analyses based on a fetal presentation at the trial of labor, we demonstrated that patients with a breech presentation, regardless of whether they had an attempted ECV, had a higher risk of cesarean delivery as well as NRFHR tracings. These findings align with existing literature that advocates for the initial trial of the ECV as a favorable approach to managing breech presentations [11, 12].

Our study has several limitations that must be acknowledged. Firstly, it is a retrospective analysis conducted at a single center, conducted on a relatively small number of women who attempted vaginal breech delivery following a failed ECV. These facts might limit the generalizability of our findings to other settings with different clinical guidelines or patient populations. Furthermore, our results apply only to patients who meet the inclusion criteria of this study.

Furthermore, the decision regarding the mode of delivery was influenced by the subjective evaluations and perspectives of different attending physicians. This variability in clinical judgment may introduce biases that affect the study’s outcomes.

Due to the retrospective nature of our study, we were limited by the number of cases available during the study period; therefore, a power analysis was not conducted before the initiation of the study.

Furthermore, the retrospective design prevented us from obtaining comprehensive data on the number of women who opted for a primary cesarean section due to breech presentation, as well as information on those who did not meet the inclusion criteria. This gap in data may impact the overall completeness of our analysis. Further research is required, ideally in the form of multicenter randomized control trials, to determine the optimal mode of delivery as well as the advantages and disadvantages of each option in patients with a breech presentation at term.

Despite the acknowledged limitations, our study offers novel and valuable insights into an increasingly common clinical scenario: women who opt for vaginal delivery despite having a fetus with a breech presentation at term, an area where data is scarce. Furthermore, we were able to compare outcomes across three clinically relevant scenarios—successful External Cephalic Version (ECV), failed ECV, and ECV not attempted—reflecting the actual choices faced by women with a breech presentation who desire a vaginal delivery at term.

Our findings indicate that a successful external cephalic version (ECV) significantly increases the likelihood of vaginal delivery. Conversely, a failed ECV attempt does not diminish the chances of vaginal delivery or increase adverse outcomes compared to women opting for breech delivery where ECV was not attempted. These results suggest that attempting an ECV is worthwhile as it will only increase the rate of vaginal delivery if successful and poses no great maternal or neonatal risk if unsuccessful.

Our study contributes to the growing body of literature supporting ECV as the recommended initial intervention for breech presentations. Importantly, our findings suggest that a failed ECV should not preclude the option of vaginal breech delivery, as it does not appear to negatively impact the likelihood of a successful vaginal birth or compromise maternal and neonatal outcomes. This highlights the importance of offering women a range of options and providing individualized care when managing breech presentations, balancing the benefits of ECV with the possibility of vaginal breech delivery.

## Conclusion

Our study suggests that for women at term with a breech presentation who are strongly motivated to pursue vaginal delivery, attempting an external cephalic version (ECV) is recommended. A successful ECV significantly enhances the likelihood of vaginal delivery. Additionally, a failed ECV attempt does not correlate with an increased risk of adverse outcomes or cesarean delivery compared to breech presentations where ECV was not attempted. This suggests that, in patients who meet the inclusion criteria of this study, attempting an ECV poses no harm and can only increase the rate of vaginal delivery if successful. These findings can provide valuable guidance for counseling women who present at term with a breech presentation and wish to avoid cesarean delivery.

## Supplementary Information

Below is the link to the electronic supplementary material.

Supplementary file (XLSX 37 KB)

## Data Availability

The dataset generated and analyzed during the current study is available as a supplementary file.
